# Cardiovascular risk in axial spondyloarthritis—a systematic review

**DOI:** 10.1007/s10067-023-06655-z

**Published:** 2023-07-07

**Authors:** Rainer Hintenberger, Barbara Affenzeller, Valeriia Vladychuk, Herwig Pieringer

**Affiliations:** 1 Department for Internal Medicine II, Kepler University Hospital GmbH, Johannes Kepler University Linz, Krankenhausstraße 9, 4020 Linz and Altenbergerstraße 69, 4040 Linz, Austria; 2 Department for Internal Medicine II, Kepler University Hospital GmbH, Krankenhausstraße 9, 4020 Linz, Austria; 3 Diakonissen Hospital Linz, Linz, Austria and Paracelsus Private Medical University Salzburg, Salzburg, Austria

**Keywords:** Atherosclerosis, Axial spondyloarthritis, Heart disease, Hyperlipidemia, Vascular disease

## Abstract

Cardiovascular manifestations are common in patients suffering axial spondyloarthritis and can result in substantial morbidity and disease burden. To give an overview of this important aspect of axial spondyloarthritis, we conducted a systematic literature search of all articles published between January 2000 and 25 May 2023 on cardiovascular manifestations. Using PubMed and SCOPUS, 123 out of 6792 articles were identified and included in this review. Non-radiographic axial spondyloarthritis seems to be underrepresented in studies; thus, more evidence for ankylosing spondylitis exists. All in all, we found some traditional risk factors that led to higher cardiovascular disease burden or major cardiovascular events. These specific risk factors seem to be more aggressive in patients with spondyloarthropathies and have a strong connection to high or long-standing disease activity. Since disease activity is a major driver of morbidity, diagnostic, therapeutic, and lifestyle interventions are crucial for better outcomes.

**Table:** 

**Key Points** • *Several studies on axial spondyloarthritis and associated cardiovascular diseases have been conducted in the last few years addressing risk stratification of these patients including artificial intelligence*. • *Recent data suggest distinct manifestations of cardiovascular disease entities among men and women which the treating physician needs to be aware of*. • *Rheumatologists need to screen axial spondyloarthritis patients for emerging cardiovascular disease and should aim at reducing traditional risk factors like hyperlipidemia, hypertension, and smoking as well as disease activity*.

**Supplementary Information:**

The online version contains supplementary material available at 10.1007/s10067-023-06655-z.

## Introduction

Cardiovascular comorbidities are common in patients with radiographic and non-radiographic (r-, nr- resp.) axial spondyloarthritis (axSpA) and known for many years, yet many aspects of these comorbidities are still under investigation. Fortunately, almost 2.5-fold more articles were published in the time period 2010–2020 compared with the period before (2000–2010) according to our search strategy (see supplemental material). In addition, gender differences indeed are gaining more and more attention in the last years but data is still scarce, although gender differences could lead to distinct manifestation, course and comorbidities of axSpA [1].

While an association between disease and cardiovascular risk was not well defined until lately, addressing risk reduction is nowadays crucial in clinical practice.

Not only the concomitant inability to move or perform sports due to pain and stiffness seems to be the culprit for increased risk of cardiovascular disease (CVD), but also independent risk factors that accompany axSpA burden including environmental factors and gender differences.

For better addressing this topic, recommendations for management of cardiovascular (CV) risk in patients with rheumatic diseases have been proposed in 2016 by the European League Against Rheumatism (EULAR), although a specific recommendation for r-axSpA and nr-axSpA respectively is still lacking [2]. Since these two entities are distinct diseases with distinct characteristics, risk assessment should be individualized. Inhibitors of Cyclooxygenase (COX)-2 for example are known to increase risk of cardiovascular events in patients suffering pain from other than rheumatologic causes but seem to reduce risk in ankylosing spondylitis (AS) patients due to a decrease in inflammatory burden and therefore overall reduction of CV risk in this vulnerable population [3, 4].

A main factor of high risk is thought to be an earlier and faster genesis of atherosclerosis similar to that seen in patients suffering systemic lupus erythematosus (SLE). As for this population, patients with AS can benefit from traditional optimization of risk factors such as lipid-lowering agents and antihypertensive medications but disease-specific thresholds for risk assessment and treatment targets are yet to be developed. This is underlined by the fact that traditional CV risk algorithms are performing poorly in a retrospective AS cohort using machine learning. In this study, the best, but also modest, performance showed the Systematic Coronary Risk Evaluation (SCORE) and the Reynold’s risk score (RRS) compared to machine learning [5]. This was also shown before for rheumatoid arthritis (RA), psoriatic arthritis (PsA), and SLE [6].

Of note, not all classic CV endpoints are of major concern in patients suffering AS, but others do pose an even higher risk. For this reason, identification of “personal” risk factors is crucial for optimal treatment and the reduction of morbidity burden. This article summarizes current knowledge and therefore aims to help in terms of identification and management of CV manifestations in AS.

## Methods

**Fig. 1:**
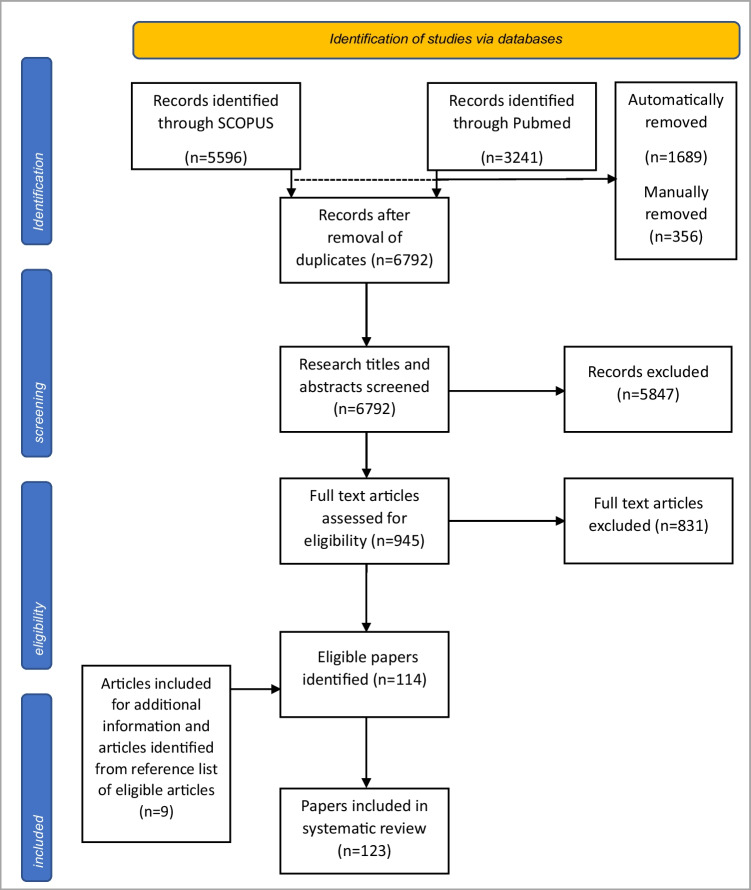
Flow diagram of identification and inclusion of papers

The extensive systematic literature search was conducted according to the recommendations of the Preferred Reporting Items for Systematic Reviews and Meta-Analyses (PRISMA) statement 2020 [7]. SCOPUS and PubMed were chosen to identify relevant literature. In total, 8837 papers were primarily identified, leaving 6792 after removal of duplicate articles. After screening of these 6792 papers, 945 were retrieved and assessed for eligibility of which 114 were included in this review. In addition, 9 articles were included for further information and articles from reference list of eligible articles. Duplicates were removed automatically as well as manually (Fig. 1). The literature search on either platforms was last updated on 25 May 2023. Meta-analysis, review articles, original articles, and case reports were included in this review. Papers published before 2000 were excluded as well as case reports with 5 or less cases.

## Results

### Epidemiology

The first 3 studies on cardiovascular mortality in ankylosing spondylitis were conducted in the late 1970s and early 1980s, respectively, showing similar results in terms of a higher risk ratio for CV mortality (about 1.3, *n* = 836) not including morbidity due to radiation therapy which was originally thought to be the main driver of increased mortality in those patients [8, 9]. In 2007, a register study from the Netherlands retrieved data from patients with inflammatory arthritis—comprising rheumatoid arthritis (RA) and AS—presented at a general practice. Patients aged 50–75 were stratified by age and sex and CVD prevalence compared to healthy controls were analyzed, resulting in an almost 1.6-fold increase in myocardial infarction and almost twofold taking cerebrovascular events into account [10]. A large study from a national insurance dataset in Taiwan identified 11,701 patients suffering AS and 58,505 controls showed similar results with significant differences in hypertension (HTN), ischemic heart disease (IHD), hyperlipidemia (HLP), congestive heart failure (CHF), and arrhythmias (ORs 1.87 [95% CI 1.75–1.99], 2.74 [95% CI 2.15–3.49], 1.46 [95% CI 1.35–1.57], 1.42 [95% CI 1.17–1.73], and 1.82 [95% CI 1.58–2.09] resp.) although this has to be interpreted with caution since relative risk strongly overrates clinical relevance when prevalence is low which was the case for IHD in this study due to a young collective. Interestingly, in this cohort, there was no sign for increased risk of stroke [11] which is contrary to more recent studies [12–14]. A recent meta-analyses including 40 studies showed similar data for hypertension (ORs 1.58 [95% CI 1.29–1.92]) and CVD (OR 1.42 [95% CI 0.999–2.03]) [15].

Although CVD is not the only cause of higher mortality in axSpA patients, this is the largest group accounting for 40% of cases followed by malignant disease (27%) according to a single-center study with 360 patients and a mean follow-up time of 31.9 years. Independent predictors of increased mortality were C-reactive protein (CRP), non-steroidal antirheumatic drugs (NSAIDs), and work disability with OR of 2.68 (95% CI 1.774–4.048), 4.35 (95% CI 1.753–10.771), and 3.65 (95% CI 1.400–9.506) resp. Diagnostic delay was not a significant risk factor probably due to lower disease and inflammatory burden and thus leading to longer time to diagnosis [16]. A recent meta-analysis showed an increased all-cause mortality RR 1.64 [95% CI 1.49–1.80] besides higher death rates from CV causes RR 1.35 (95% CI 1.01–1.81) in 6 and 3 studies respectively [17].

Of note, a Chinese study on life expectancy showed a mean loss of 7.0 years in male patients and 1.2 years in female patients who died between 1999 and 2008 compared to the general population. Although life expectancy of the general population could not be ascertained by the article [18], there are several studies regarding cardiovascular mortality with similar death rates [19], among other articles, one study with 166,920 patient years also identified male gender as independent risk factor for vascular death (HR 1.46 [95% CI 1.13–1.87]). Interestingly, in this study, lack of exposure to NSAIDs was also an independent risk factor in patients aged 65 year or older [20].

In a prospective cohort study with 6448 AS patients and stringent inclusion parameters, the overall hazard ratio (HR) for acute coronary syndrome (ACS), stroke, and venous thromboembolism (VTE) was 1.54 (95% CI 1.31–1.82), 1.25 (95% CI 1.06–1.48), and 1.53 (95% CI 1.25–1.87), respectively. Female patients showed more incident VTE than male patients (HR 1.82 [95% CI 1.28–2.58] vs. 1.41 [95% CI 1.10–1.80]) and ACS vice versa (HR 1.21 [95% CI 0.76–1.92] vs. 1.54 [95% CI 1.33–1.90]) [12]. A recent meta-analysis showed a similar risk especially for ischemic stroke (HR 1.46, 95% CI 1.23–1.68) [21].

Vascular pathologies in terms of aortitis as well as valvular pathologies and conduction abnormalities are also part of the spectrum of comorbidities [22] and are discussed below.

### Traditional CV risk factors

#### Atherosclerosis

Subclinical atherosclerosis is a well-known manifestation of axSpA and a foundation for several CV morbidities. Recent data showed no significant difference in atherosclerotic burden between AS and nr-axSpA; hence, nr-axSpA, which are underrepresented in axSpA studies, have probably the same risk for generation of subclinical atherosclerosis. This finding was independent of CRP levels in this cross-sectional study with 806 patients including 21% nr-axSpA patients. Regardless of elevation of CRP, ASDAS and bath ankylosing spondylitis disease activity index (BASDAI) was associated with presence of carotid plaques [23]. These findings are supported by a study of 67 AS patients with low disease activity (BASDAI < 4) that did find no difference in intima-media thickness, plaques, and pulse wave velocity in AS and matched individuals that presented the same traditional risk factors [24]. Of note, 40% of patients had to be reclassified to high-risk patients after plaques were recognized in carotid ultrasound [23]. In contrast, CV events are indeed associated with persisting elevation of CRP through clinical visits [25]. Also, radiographic progression of the spine is accompanied by raised markers of subclinical atherosclerosis [26]. Women with high-risk SCORE showed higher plaque burden and disease activity compared to men in a cross-sectional Spanish study of 611 men and 301 women [27].

Blocking tumor necrosis factor (TNF)-a not only reduces disease activity but also seems to be protective of intima-media thickening according to studies that included more than 50% of patients treated with TNF-a blockade [28].

Alarmingly, computed coronary tomography showed significantly higher atherosclerotic lesions in young AS patients with a mean disease duration of 10 years compared to morbidity-matched controls [29].

A couple of biomarkers have been introduced as potential players in the pathogenesis of atherosclerosis in axSpA [30, 31]. Among these, interleukin-17 and its pathway, respectively, play an important role in pathogenesis of SPA but additionally in vasculitis and atherosclerosis. An extensive review on this topic has been published recently [32]

#### Hypertension

Surprisingly, there is little but conflicting data regarding the prevalence of hypertension in axSpA patients, yet, several studies showed higher risk for hypertension among AS patients [8,33] and a high risk of organ damage [34, 35]. In fact, longer disease duration—especially more than 5 years—and delay in diagnosis seem to be major risk factors for hypertension according to a large (*n* = 413, male = 77.2%) longitudinal cohort study from Shi et al. Additionally, recurrent rise in erythrocyte sedimentation rate (ESR) was also associated with hypertension highlighting the role of inflammation in developing hypertension. Interestingly, NSAIDs did not lead to a higher incidence of hypertension in these patients but salazopyrin (SZP) did [35].

An interesting retrospective cohort study conducted by Chou et al. showed that comorbidities, namely HTN and diabetes mellitus, did not only occur more often in the AS cohort but also had a higher impact on leading to the outcome measure ACS, especially when both risk factors combined were present [36]. Lately, a large cohort (*n* = 1111) with AS patients who are suffering uveitis were shown to have higher mortality rates after adjustment for age, sex, and comorbidities compared to AS patients without uveitis [37].

Furthermore, prevalence of hypertension seems to rise with increasing radiographic progression [38]. Another study showed an association between hypertension and longer disease duration as well as with pure axial involvement, but there was no association with ischemic heart disease, stroke, diabetes mellitus, or dyslipidemia [39].

Pathogenic mechanisms have been proposed in the last few years. Damage-associated molecular patterns acting as T-like receptor ligands are leading to activation of Th1 and Th17 cells. The activation might lead to the production of reactive oxygen species and interleukin-17 with the abovementioned involvement in vascular damage. Leptin, homocysteine production, and sodium retention also play a part in development of hypertension [40].

#### Hyperlipidemia

As with hypertension, the inflammatory state has an utmost influence on dyslipidemia. Hypertriglyceridemia and athrogenic index were significantly higher than in the control group [41]. Surprisingly, implementation of TNF-a-blockade prescription led to higher total cholesterol levels, high- and low-density lipoproteins, triglycerides, and higher atherogenic index [42] but this effect was not seen after a 2-year follow-up period [43]. A recent meta-analysis on lipids in axSpA showed highly significant reduction in high-density lipoprotein in axSpA patients compared to healthy controls [44]. Interestingly, levels of lipoprotein(a) which were believed to be almost exclusively genetically determined were shown to be decreased already after 6 weeks and 6 months of treatment with either MTX alone or combined with TNF-a-blockade or TNF-a-blockade monotherapy in a cohort comprising AS and PsA (*n* = 25, *N* = 37 resp.) despite the small case number [45].

#### Diabetes

Diabetes mellitus type II (DM2) in AS patients has a more deleterious effect on myocardial infarction, stroke, and all-cause mortality compared to patients suffering DM2 alone (HR 1.62 [95% CI 1.16–2.27], 2.27 [95% CI 1.78–2.88], and 1.34 [95% CI 1.09–1.66] resp.) [46].

#### Metabolic syndrome

AS patients have significantly higher prevalence of metabolic syndrome (MetS). MetS itself increased the 10-year CVD risk according to a small study with 63 men [47]. Furthermore, MetS is common in r-axSpA as a Spanish study showed (33% of patients). For identifying metabolic syndrome, arthrogenic index seems to be a potential prediction marker [48].

### Heart disease

Several cardiac manifestations of SPA have been identified which are important reasons for progressive morbidity. There is evidence due to a present case–control study, that patients with AS and without cardiovascular risk factors have a higher prevalence for left ventricular systolic and diastolic dysfunction compared to the healthy control group. The second common cardiovascular risk factors based on echocardiography and ECG are left anterior fascicular block, left-axis deviation, and aortic valve insufficiency [49].

Aortic valve insufficiency is one of the most important cardiovascular involvements occurring in 18% of patients [50]. It has been proposed to be a result of chronic aortitis which involves the aortic root and leads to a dilatation and insufficiency [51, 52]. Especially for HLA-B27-positive male patients, an increased aortic root index—but no difference in the prevalence for aortic valve regurgitation compared with HLA-B27-negative patients—was found. Nevertheless, echocardiographic monitoring should be considered regardless of HLA-B27 status in male patients [53]. In general, echocardiography, cardiac magnetic resonance imaging (MRI), and computed tomography (CT) scans can be tools for follow-up [54]. To evaluate aortic regurgitation or conduction abnormalities, electrocardiogram and echocardiography are recommended as routine tools because their symptoms alone are not very specific and straightforward to interpret [55]. Searching for cardiovascular risk factors at least once every 5 years is also noted in the EULAR recommendation update 2015/2016 [2].

In addition, rheumatologists should be aware of cardiac rhythm disturbances in patients suffering SPA although there is contrary data on this topic. Based on a nationwide cohort study from Sweden from 2006 to 2012 including 6448 patients, the most common cardiac rhythm disturbance were atrioventricular (AV) blockades II–III, atrial fibrillation (AF), and aortic regurgitation (AR) compared to the general population with age- and sex-adjusted HR of 2.27 (95% CI 1.59–3.26), 1.35 (95% CI 1.16–1.57), and 1.93 (95% CI 1.28–2.91), respectively [52]. Furthermore, another study from South Korea with a similar sample size showed significant difference only for AF and AR (HR 2.55 [95% CI 1.49–4.37] and 1.20 [95% CI 1.04–1.39] resp.), but not for AV blockades in these multivariate analyses. Follow-up time was 6 years in the former study and thus 4 years shorter than the latter one with similar mean age of included patients [56]. In contrast, a study of 100 patients with long-standing disease (mean disease duration of 33 years) and older age (mean 54.9 years) showed no significant differences in conduction abnormalities and only a trend towards aortic and mitral regurgitation [57].

The fact that AS is an independent risk factor for AF especially in younger patients (< 40 years) was again recently shown. However, this risk could just be reproduced for male patients. Current TNF-a inhibitor therapy did increase the risk about 3 times that of patients without biological therapy in this study. Whether this is due to higher disease activity that made biologic therapy necessary or an effect of TNF-a is still a matter of debate [58]. Conversely, a biobank study from the UK did show that only women are at greater risk for AF (HR 1.53, 95% CI 1.13–2.07) [59].

Atrioventricular (AV) re-entry tachycardia occurs more often in AS patients; the pathogenesis can be found in inflammatory processes and fibromuscular proliferation [60]. Particularly, AS patients with symptoms like palpitation, dizziness, dyspnea, or syncope should undergo ECG and electrophysiological examinations to check for paroxysmal AV nodal re-entry tachycardia and Wolff-Parkinson-White syndrome [61].

The aim of several studies was to determine electrocardiographic parameters to identify serious life-threatening arrhythmias in SPA patients. T-peak to T-end interval and its relation to corrected QT time, obtained by Holter electrocardiogram, is a known marker for disrupted ventricular depolarization possibly leading to malignant ventricular arrhythmias and therefor aim of a cross-sectional study that examined 76 patients without comorbidities influencing the autonomous nervous system. Patients with AS are more likely to have disrupted ventricular depolarization compared to healthy controls [62, 63]. It is important to determine risk groups in the AS patients which are more likely to develop cardiac conduction disturbance. Especially patients with a higher ASDAS-CRP, a history of anterior uveitis, and longer duration of the disease measured by the age at diagnosis are suggested to have a higher risk [64, 65].

Routine echocardiography seems to be crucial to find prognostic parameters for patients with axSpA. A depressed longitudinal strain in combination with a high-sensitive troponin I (hsTnI) ≥ 3.0 pg/ml can predict MACE in this population [66]. Generally, there is evidence that patients with axSpA have an impaired left ventricular longitudinal strain and a higher risk for diastolic dysfunction measured with the E/E′ ratio than the control groups after adjustment for confounding factors [67]. Diastolic dysfunction itself occurs more often in patients with a long-standing disease [57]. A difference exists when comparing patients with AS to nr-axSpA. The global longitudinal peak systolic strain as a marker for subclinical myocardial dysfunction is lower in patients with AS [68]. Another interesting study showed an association with endophtalmitis and new onset of myocardial infarction [69].

### Macrovascular disease

An association of AS and inflammation of large vessels has been demonstrated in several case reports and articles [70–72]. Mainly, association with Takayasu arteriitis (TAK) is described in the literature. Large vessel involvement in patients suffering SPA leads to pronounced morbidity. Unfortunately, a specific therapy is not available at this moment [73]. Interestingly, one recent study found signs of axSpA and inflammatory bowel disease in 4 of 34 patients with formerly diagnosed TAK proposing screening for SPA whenever diagnosis of “primary” TAK is made [74]. Of note, in contrast to idiopathic or other secondary large vessel vasculitis, SPA-associated vasculitis seems to primarily become symptomatic in spring [73]. Additionally, high inflammatory markers and type IIb vascular involvement are associated with peripheral and axSpA [70, 75].

Two big Spanish cross-sectional projects including patients from 28 primary care centers showed significant association with peripheral artery disease, but patient collective was comprised of “inflammatory polyarthropathies” and “spondylopathies” after the respective ICD-10 code [76].

### Impact of lifestyle

Changing lifestyle habits that have negative impact on axSpA and CV risk does pose an important role in attenuating disease manifestations of axSpA but also associated CVD. For this reason, EULAR has proposed recommendations for lifestyle behaviors in 2021 [77].

Especially, exercise is an important tool to improve disease-related outcomes; however, many patients do not frequently exercise due to different reasons including fatigue and tiredness [78], which reflects the lower peak oxygen uptake as a marker of cardiorespiratory fitness [79]. A single blinded randomized controlled trial (RCT) showed effect on arterial stiffness and pulse wave velocity (PWV) already after 12 weeks of combined endurance and strength training despite the small number of cases (*n* = 24) [80]. Furthermore, cardiorespiratory fitness was inversely correlated with arterial stiffness similar to the general population but independent from traditional risk factors [81]. High-intensity exercise for 3 months was shown to reduce AS disease activity scale (ASDAS) by 0.6 in 3 months according to another study conducted in 2020. In a retrospective study on 24 patients with limited spinal movement due to AS, two weeks of guided Yoga decreased systolic blood pressure and heart rate significantly [82].

Smoking is a major driver of inflammation and clinical activity among axSpA patients. Additionally, smoking can per se enhance physical inactivity via its negative effect on lung function [83, 84]. Nevertheless, the prevalence of smoking is much higher in 2 SPA cohorts compared with the UK population in 2018 (24–29% vs. 15%). In general, there are many negative aspects of smoking for axSpA but there are also many confounding factors that make calculation of smoking as an independent risk factor difficult [85]. No study found in our literature search evaluated smoking as an independent risk factor for CVD in axSpA.

There is no evidence of specific dietary regimens to help improve cardiovascular risk in axSpA. Yet, the Mediterranean diet has shown to be beneficial for both disease activity and cardiovascular health in the general population [86]. Therefore, the Mediterranean diet could be recommended to axSpA patients.

### *Impactof therapeutic interventions*

While there is a lot of evidence which argues for an increased CV risk in SpA patients, there is much less robust data that could help to estimate the effect of anti-inflammatory therapy on CV outcome in this population. A list of included studies assessing interventions to lower CV risk is provided in Table 1.

**Table 1:** Studies assessing cardiovascular outcomes after specific interventions

Author, year of publication	Disease (*n*) classification criteria	Study design	Females	Intervention	Outcome (*p* < 0.05) or (95% CI)
Sveaas SH et al. 2014 [80]	axSpA (28) ASAS + BASDAI > 3.5	Single blinded randomized controlled pilot study	50%	Endurance and strength exercise 12w vs. usual treatment Aix, PVW: 2^nd^ outcome measure	AIx (%): − 5.3 (− 11.0, − 0.5). PVW (m/s): − 0.3 (− 0.7, 0.0)
Berg IJ et al. 2018 [81]	AS (118)	Cross-sectional cohort study	36%	VO2peak via treadmill test	AIx and PWV inversely associated with VO2peak CRP and ASDAS no association with VO2peak
Singh et al., 2021 [82]	AS (24)	Retrospective analysis	N/A	2w Yoga retreat	Systolic bp↓ (% change 6.22 mmHg)
Syngle A et al. 2013 [89]	AS (20) mNYc + BASDAI ≥ 4	Prospective, controlled, open-label	25%	Spironolacton 2 mg/kg/day	FMD↑, nitrite↓, CRP↓, ESR↓
Garg N et al. 2021 [90]	AS (40) mNYc + BASDAI ≥ 4	RCT	38%	Olmesartan 10 mg/day	FMD↑, EPC↑, nitrite↓, CRP↓, ESR↓, IL-6↓, TNF-a↓, VCAM-1
Garg N et al. 2015 [88]	AS (32) mNYc	Single-blind, placebo-controlled, parallel study	67%	Rosuvastatin 10 mg daily	FMD↑, IL-6↓, TNF-a↓
Oza A et al. 2017 [91, 92]	AS	Incident user cohort study	21%	Statin use	37% reduction all-cause mortality
Tsai WC et al. 2015 [4]	AS (10763) ICD-9-CM code	Case–control study	54,9%	NSAIDs exposure and risk of MACE	OR 0.23; 95% CI 0.07 to 0.76 MPR < 80% for 6 months: OR 1.41; 95% CI 1.07 to 1.86
Wu LC et al. 2016 [112]	AS (4829) ± CAD ICD-9-CM code	10-year population-based case–control study	~ 44%	High cumulative dose celecoxib (> 300 mg/day) High cumulative dose SZP (≥ 1000 mg/day)	CAD risk OR 0.34; 95% CI, 0.13 0.89 CAD risk OR 0.63; 95% CI, 0.40–0.99
Tam HW et al. [111]	AS (1208) ICD-9-CM code	10-year population-based retrospective cohort study	40%	High cumulative dose celecoxib (> 300 mg/day) High cumulative dose SZP (≥ 1000 mg/day)	CVD risk HR = 0.39; 95% CI 0.20–0.77 HR = 0.72; 95% CI = 0.58–0.91
Kiortsis DN et al. 2006 [104]	RA (50) + AS (32)	Prospective design	48% AS (3%)	IFX iv (6 months) ± NSAIDs, ± SZP, ± MTX	TC ↑, TG ↑ HDL, LDL, TC/HDL ratio + TG/HDL ratio no change
Van Eijk et al. 2009 [102]	AS (92) mNYc BASDAI ≥ 4	Prospective design	36%	ETN (3 months)	ESR↓, CRP↓, SAA↓, TC↓, HDL↓, LDL↓, Trigl.↓, ApoA-I↓
Syngle A et al. 2010 [96]	AS (12) mNYc BASDAI ≥ 4	Prospective, uncontrolled, open-label	20%	IFX 5 mg/kg single iv + SZP + NSAIDs	FMD↑, nitrite↓, CRP↓
Mathieu S et al. 2010 [101]	AS (34) mNYc	Prospective design	N/A	IFX (59%), ETN (21%), ADA (21%)	CRP↓, ESR↓, Chol↑, HDL↑
Angel K et al. 2012 [93]	RA + AS + PSA (55); AS (19)	Prospective, not randomized	67%	1 yr anti-TNF-a	PWV↓, cIMT↓, CRP↓, ESR↓, Calp↓
Mathieu S et al. 2013 [99]	AS (49) mNYc	Prospective, not randomized	39%	ETN (53%), ADA (35%), IFX (12%)	CRP↓, ESR↓ PWV, AIP no change
Genre F et al. 2015 [100]	AS (30) mNYc	Prospective design	30%	IFX single infusion	E-selectin, VCAM-1 (120 m after admin)
Lee JL et al. 2018 [114]	RA (3167) + AS (561) + PsA (412)	Prospective National Cohort Study	66.4%	Current anti-TNF-a therapy	HR 0.85, 95% CI 0.76–0.95
Knyazeva LA et al. 2019 [95]	AS (42) mNYc	Prospective design	33%	2 yr GOL	cIMT↓,AIP↓, stiffness↓
Vegh E et al. 2020 [43]	RA + AS (53); AS (17)	Prospective, not randomized	21%	1 yr anti-TNF-a ETN (37) or CZP (16) ± MTX (31)	PWV↓, cIMT↓, CRP↓
Min HK et al. 2020 [43]	axSpA (238) mNYc or ASAS	Prospective longitudinal cohort study	26%	TNF (132) vs. non-TNF (106) ADA (36%), IFX (33%), ETN (30%)	TC↑ LDL-C, HDL-C, AIP no change
Kwon OC et al., 2022 [115]	axSpA (450)	Retrospective cohort study	24%	anti-TNF-a exposed (multivariate analysis)	CVD risk HR 0.30, 95% CI 0.11–0.87
Fakih O et al. 2023 [108]	AS (22.929) ICD-10 code	National cohort study	55%	NSAIDs, csDMARDs, anti-TNF-a, anti-IL-17	NSAIDs: SHR 0.39; 95% CI 0.32–0.50 Anti-TNFa: SHR 0.61; 95% CI 0.46–0.80

*Abbreviation:ADA*, Adalimumab; *AIP*, atherogenic index of plasma; *AIx*, augmentation index; *Apo-A*, apolipoprotein A; *AS*, ankylosing spondylitis; *ASAS*, Assessment of SpondyloArthritis International Society; *ASDAS*, Ankylosing Spondylitis Disease Activity Score; *axSpA*, axial spondylarthritis; *BASDAI*, Bath Ankylosing Spondylitis Disease Activity Index; *BASMI*, Bath Ankylosing Spondylitis Metrology Index; *BP*, blood pressure; *Calp*, Calprotectin; *CVD*, cardiovascular disease; *cIMT*, carotid intima media thickness; *CZP*, Certolizumab; *CI*, confidence interval; *csDMARDS*, conventional synthetic disease modifying antirheumatic drug; *CAD*, coronary artery disease; *CRP*, C-reactive protein; *EPC*, endothelial progenitor cells; *ESR*, erythrocyte sedimentation rate; *ETN*, Etanercept; *FBG*, fasting blood glucose; *GOL*, Golimumab; *HR*, hazard ratio; *HDL*, high density lipoprotein; *FMD*, low-mediated dilation; *MACE*, major adverse cardiac event; *IFX*, Infliximab; *IL*, interleukin; *LDL-C*, low density lipoprotein; *MPR*, medication possession rate; *MTX*, Methotrexat; *mNYc*, modified New York criteria; *NSAIDs*, non steroidal anti inflammatory drugs; *OR*, odds ratio; *VO2peak*, peak oxygen uptake; *PsA*, psoriatic arthritis; *PWV*, pulse wave velocity; *RA*, rheumatoid arthritis; *SZP*, Salazopyrin; *SHR*, subhazard ratio; *TC*, total cholesterol; *TG*, triglycerides; *anti-TNFa*, Tumor necrosis factor-alpha inhibitors; *VCAM-1*, vascular cell adhesion molecule-1

As a first step, however, before a specific anti-rheumatic treatment is prescribed, the importance of assessing the CV risk in SpA patients should be emphasized and—if appropriate—interventions as those recommended for the general population should be considered. This is also emphasized in the 2015/2016 updated EULAR CV risk management recommendation, which clearly state that rheumatologists are responsible for CV risk management in patients with inflammatory joint disorders, including those with AS [2]. Beside anti-inflammatory treatment, the key risk factors cholesterol, blood pressure, cigarette smoking, diabetes, and adiposity should deserve attention also in axSpA patients [87]. In this context, it is of interest that small studies suggest positive effects of statins [88], spironolactone [89], and angiotensin receptor blockers in AS patients [90]. In a mixed population, rosuvastatin was associated with regression of carotid plaques [91] and UK data shows a reduced mortality in AS patients using statins [92].

There is some evidence that anti-rheumatic treatment in axSpA patients might also yield some beneficial effects regarding CV outcomes. Some—mostly small- and short-term—studies, often in a mixed population, have found favorable effects of biologic disease-modifying anti-rheumatic drugs (DMARDs) on surrogate markers of CV risk such as markers of endothelial function or arterial stiffness [93–97], while others did not [98, 99]. TNF inhibitors seem to reduce biomarkers of endothelial cell activation in AS patients [100]. However, it is not fully clear whether the improvement of vascular function will finally translate into an improved CV outcome.

In addition, several studies investigated the effect of TNF inhibitors on the lipid profile in axSpA patients [43, 101–104]. In summary, besides a reduction of markers of inflammation, most of these studies found an increase in total cholesterol and HDL levels; however, total cholesterol to HDL ratio was stable or improved and the lipid profile was found to be less pro-atherogenic.

One of the cornerstones of therapy in axSpA patients are NSAIDs. It is well established that NSAIDs are associated with an increased number of CV events in the general population [105, 106]. For axSpA patients, this association is much less clear, as there are reports that suggest a possible beneficial effect of NSAIDs with regard to CV events in axSpA patients [4]. In a recent systematic review of observational studies, Karmacharya et al. did not find an increased CV risk for NSAIDs as well as cyclooxygenase 2 (COX2) inhibitors in AS patients. For the whole group, the risk of a CV event showed an RR of 0.96 (95% CI 0.51–1.81). The risk of a cerebrovascular accident was significantly lower in NSAID users (RR 0.58 [95% CI 0.37–0.93]). COX2 inhibition use was associated with a reduced risk of all CV events (RR 0.48 [95% CI 0.33–0.70]) [107].

Additionally, a recent national cohort study (*n* = 22,929) from France showed a significant reduction of MACE in 8-year cumulative incidence after treatment with NSAIDs und TNF inhibitors, but not after IL17-inhibition or treatment with conventional DMARD_S_ [108]. However, there are also data which demonstrate an increased risk of new-onset hypertension in AS patients with continuous use of NSAIDs (HR 1.12 [95% CI 1.04–1.20]). Given these findings, there is ongoing debate about the place of NSAIDs in the context of AS and CV risk [109, 110].

One Asian retrospective cohort study found a borderline reduced CV event rate with sulfasalazine in AS patients (HR 0.65 [95% CI 0.43–0.998]) [111]. In contrast, in a population-based case–control study from Taiwan, sulfasalazine was—again borderline–negatively associated with the development of coronary artery disease (OR 0.63 [95% CI, 0.40–0.99]) [112].

Chan et al. assessed the impact of TNF inhibitors on the increased CV risk in SpA patients. The authors used a cohort of SpA patients (including 19.4% with psoriatic arthritis; overall 67.9% fulfilling AS criteria) and a matched cohort of patients with non-specific back pain. As expected, SpA patients had a higher risk of MACE (HR 1.70 [95% CI 1.29–2.26]) and cerebrovascular events (HR 1.50 [95% CI 1.08–2.07]). Of interest, SpA patients receiving treatment with TNF inhibitors (*n* = 649) had a reduced risk of MACE (HR 0.37 [95% CI 0.17–0.80]) and cerebrovascular events (HR 0.21 [95%CI 0.06–0.78]) compared with SpA patients without this treatment. Of note, there was no association between CV risk and synthetic DMARD use [113]. In a study using the Australian Rheumatology Association Database, which included patients with RA, PsA, and AS, the use of TNF inhibitors was associated with a reduced risk of CV events (HR 0.85 [95% CI 0.76–0.95]) and there was no difference between RA patients (the largest group) and AS patients (HR 1.14 [95% CI 0.96–1.36]). Of note, patients who had stopped biological DMARDs did not show this reduction of CV events (HR 0.96 [95% CI 0.83–1.11]) [114]. In a Korean retrospective cohort study, the authors found a reduced risk of CV events in patients receiving TNF inhibitors in unadjusted analysis and after adjustment for traditional CV risk factors, but not after further adjustment in different statistical models [115].

At present, there is an intense discussion on safety issues of JAK inhibitors, which also play a role in the treatment of axSpA. As discussed previously [116], a randomized trial in RA patients found an increased CV risk for tofacitinib as compared to etanercept or adalimumab [117]. This has been reflected in the 2022 updated EULAR RA recommendations, which caution the use of JAK inhibitors in RA patients with increased CV risk [118]. In contrast, this restriction has not been incorporated in the 2022 EULAR axSpA recommendations [119]. A recent meta-analysis comprising 19 RCTs investigating the effect of upadacitinib (UPA) on lipids and cardiovascular events showed no increase in cardiovascular events but an increase in LDL-C and HDL-C leaving the ratio unchanged although follow-up was only 52 weeks [120]. While more data are expected to finally clarify the role of JAK inhibitors with regard to CV risk, a prudent approach seems advisable when JAK inhibitors are considered for SpA patients at increased CV risk [121].

To date, no data with CV outcome have been published for IL-23 inhibitors.

## Discussion

Our review gives a comprehensive overview on cardiovascular manifestations in patients suffering axSpA. While some traditional risk factors do not occur more often in axSpA, others are driving morbidity even more compared to the general population. On the one hand, small sample numbers in studies especially in nr-axSpA make substantial risk stratification difficult. On the other hand, estimating the CV risk for these patients seems to be of utmost importance. For that reason, a lot of effort was put into developing risk-identifying parameters or algorithms to supersede old traditional scoring methods that are known to perform poorly in axSpA patients. This is most likely explainable with a different pathomechanism leading to the same or even higher CV risk compared to patients with similar risk but without axSpA. Some studies used machine learning methods to find prediction models with variable success which have not yet been incorporated into formal recommendations [5]. To some extent, ultrasound of carotid arteries, echocardiography, and coronary CT scans can identify patients at risk similar to medical checkups performed in the general population potentially leading to stricter optimization of modifiable risk factors. Also gender-specific differences and characteristics of comorbidities should be taken into account. However, many of the cited studies on CV risk in axSpA have small sample sizes and inhomogeneous collectives. This does not only account for population age but also gender, which per se affects the underlying CV risk. Many studies included patients with axSpA, but also with similar inflammatory arthropathies. This limits the specificity of the results of these studies. In addition, the classification as well as our view of axSpA has changed over time. This is probably best reflected by the introduction of the concept of non-radiographic axSpA. It is likely that these patients behave similar, but not identical as compared to classical AS.

Of note, involvement of potentially life-threatening manifestations of CVD in axSpA patients with sudden occurrence should be paid particular attention to. Therefore, some articles have been published regarding this topic mentioned above.

Since inflammatory burden is strongly connected to CV risk, lowering especially axial disease activity is crucial for also reducing major cardiovascular endpoints and mortality. The best evidence for reduction of disease and inflammatory burden has been shown for TNF inhibitors, although other conventional DMARDs showed at least a small effect. Regarding inhibition of Janus kinases, the last word has not yet been spoken; whether this substance group is intrinsically increasing CV risk or risk is just reduced by other biological therapies as shown for RA leading to a relatively increased risk compared to other therapies is still under debate [121]. For this reason, rheumatologists and other prescribing specialists are encouraged to exercise particular diligence when choosing Janus kinase inhibitors for therapy in patients already at risk [118].

## Conclusion

CVD is a frequent comorbidity in axSpA patients and patients with axSpA have a higher mortality because of CVD, which is the most frequent cause followed by malignant diseases. While aiming for low disease activity or—at best—remission as one of the core competencies of rheumatologists, management of axSpA patients should also include CV risk assessment. Recognizing patients at risk, especially early in the disease course, offers the possibility of timely intervention and, in turn, reduced CV morbidity and mortality later in the disease course.

More studies are needed to detect patients at risk early and prevent major disease burden. Especially, studies on major cardiovascular endpoints after pharmaceutical interventions and studies on gender differences are urgently needed to improve care of these vulnerable patients since respective data is scarce.

### Supplementary Information

Below is the link to the electronic supplementary material.

Supplementary file1 (PDF 180 KB)
