# Supplementary material for: Cardiovascular risk in axial spondyloarthritis—a systematic review
Source: Clin Rheumatol. 2023 Jul 7;42(10):2621–33. doi: 10.1007/s10067-023-06655-z (PMC10497445; doi:10.1007/s10067-023-06655-z)
Supplement: Supplementary file 1 — Supplementary file1 (PDF 180 KB) [file 10067_2023_6655_MOESM1_ESM.pdf]

APPENDIX

Cardiovascular risk in axial spondyloarthritis – A systematic review

Rainer Hintenberger[1], ORCID: 0000-0002-5142-0860, e-mail: rainer.hintenberger@kepleruniklinikum.at

Barbara Affenzeller[1], ORCID: 0009-0009-0991-1189, e-mail: barbara.affenzeller@kepleruniklinikum.at

Valeriia Vladychuk[2], ORCID: 0009-0002-6051-0726, e-mail: valeriia.vladychuk@kepleruniklinikum.at

Herwig Pieringer[3], ORCID: 0000-0003-0401-7849, e-mail: herwigpi@yahoo.com

[1] Department for Internal Medicine II, Kepler University Hospital GmbH, Johannes Kepler University Linz, Krankenhausstraße 9, 4020 Linz and Altenbergerstraße 69, 4040 Linz, Austria

[2] Department for Internal Medicine II, Kepler University Hospital GmbH, Krankenhausstraße 9, 4020 Linz

[3] Diakonissen Hospital Linz, Linz, Austria and Paracelsus Private Medical University Salzburg, Salzburg, Austria

Corresponding author: Rainer Hintenberger

Research question:

In what way are cardiovascular diseases associated with axial spondyloarthritis?

PUBMED:

Concept 1: cardiovascular diseases

Keywords:

cardiovasc*[tw] OR cardiac[tw] OR vasc*[tw] OR cv[tw] OR cardiovascul*[tw] OR “coronary heart”[tw] OR ischemic[tw] OR ischaemic[tw] OR “coronary arter*”[tw] OR “peripheral arter*”[tw] OR cerebrovasc[tw] OR stroke[tw] OR “myocardial infarction”[tw] OR “heart attack”[tw] OR pad OR “pulse wave velocity*”[tw] OR “pulse wave analysis”[tw] OR blood pressure[tw] OR hypertension[tw] OR lipids[tw] OR cholesterol[tw] OR triglycerid*[tw] OR “high density lipoprotein” [tw] OR hdl[tw] OR “low density lipoprotein”[tw] OR “lipoprotein{a}”[tw] OR "remnant cholesterol"[tw] OR “dyslipidemia” OR smoking[tw] OR “augmentation index”[tw] OR “pulse wave velocity”[tw] OR “pulse wave”[tw] OR “brachial ankle index”[tw]

MeSH:

“Heart Disease Risk Factors”[Mesh] OR “Cardiovascular Diseases”[Mesh] OR “Myocardial Infarction”[Mesh] OR “heart failure” [Mesh] OR “heart valve diseases” [Mesh] OR “myocardial ischemia” [Mesh] OR “rheumatic heart disease” [Mesh] OR hypertension [Mesh] OR “metabolic diseases” [Mesh] OR “peripheral vascular diseases” [Mesh] OR vasculitis [Mesh] OR “hemostatic disorders” [Mesh]

-AND-

Concept 2: axial Spondyloarthritis

Keywords:

“ankylosing spondylitis”[tw] OR “spondylitis”[tw] OR “spondyloarthropath*”[tw] OR “spondylarthropath*”[tw] OR “spondylitis ankylos*”[tw] OR axSPA[tw] OR r-axSPA[tw] OR nr-axSPA[tw] OR sacroileitis[tw] OR sacroiliitis[tw] OR spondyloarthrit*[tw] OR spondylarthrit*[tw]

MeSH: "Spondylitis, Ankylosing"[Mesh] OR "Axial Spondyloarthritis"[Mesh]

SCOPUS:

Concept 1: cardiovascular diseases

Keywords:

"ankylos* W/2 spondyl*" OR spondylitis OR spondyloarthropath* OR spondylarthropath* OR *axSPA OR sacroil?itis OR spondyloarthrit* OR spondylarthrit*

-AND-

Concept 2: axial spondyloarthritis

Keywords:

cardiovasc* OR cardiac OR vasc* OR cv OR cardiovascul* OR {coronary heart} OR ischemic OR isch?emic OR "coronary arter*" OR "peripheral arter*" OR cerebrovasc* OR stroke OR {myocardial infarction} OR {heart attack} OR pad OR "pulse wave velocit*" OR {pulse wave analysis} OR {blood pressure} OR hypertensi* OR lipids OR cholesterol OR triglycerid* OR "high?density?lipoprotein" OR hdl OR "low?density?lipoprotein" OR "lipoprotein?a*" OR "remnant cholester*" OR dyslipidemia OR smoking OR {augmentation index} OR {pulse wave velocity} OR {pulse wave} OR {brachial ankle index}
